# Comparison of allo-SCT, auto-SCT and chemotherapy for the treatment of patients with low- or intermediate-risk acute myeloid leukemia: a network meta-analysis

**DOI:** 10.1186/s13287-024-03766-5

**Published:** 2024-05-31

**Authors:** Wu Ye, Xia Wu, Ruying Zhao, Xuelian Jin, Hui Li, Ying Qu, Jie Ji, Zhigang Liu

**Affiliations:** 1 Department of Hematology, West China Hospital, Sichuan University, No.37 GuoXue Xiang, Chengdu, 610041 Sichuan Province China; 2 Laminar Air-flow Research Unit of Clinical Trial Center, West China Hospital, Sichuan University, No.37 GuoXue Xiang, Chengdu, 610041 Sichuan Province China

**Keywords:** Acute myeloid leukemia, Allo-SCT, Auto-SCT, Chemotherapy, Network-comparison

## Abstract

**Background:**

The therapeutic status of allogeneic stem cell transplantation (allo-SCT) as a post-remission treatment for patients with high-risk acute myeloid leukemia (AML) was well-accepted. However, the optimal treatment for patients with low/favorable- or intermediate-risk AML who achieve complete remission has remained controversial. Therefore, we conducted a network meta-analysis to discuss this disputed problem.

**Methods:**

We compared the effects of treatment strategies including allo-SCT, autologous stem cell transplantation (auto-SCT) and consolidation chemotherapy (CT) for patients with low/favorable- or intermediate-risk AML. The pooled HRs and 95% CIs for overall survival and disease-free survival were estimated with Stata12 and R software. Thirty clinical studies with 6682 patients were included in the meta-analysis.

**Results:**

The results indicated that the treatment outcome of allo-SCT was the best, followed by auto-SCT, and CT was likely the worst in the total AML patients. In patients with low/favorable-risk AML, the treatment outcome of auto-SCT was likely ranked first, followed by allo-SCT, and CT was the worst. In patients with intermediate-risk AML, the treatment outcome of haploidentical stem cell transplantation (haplo-SCT) was the best, followed by allo-SCT (excluding haplo-SCT), and auto-SCT and CT were the worst. However, the median age of the haplo-SCT group was much younger than that of the control group, which may be one of the reasons for the better prognosis of the haplo-SCT group.

**Conclusions:**

Patients with low/favorable- and intermediate-risk (non-high-risk) AML should prioritize allo-SCT if they are eligible for transplantation, and auto-SCT is optional. However, in the subgroup analysis, auto-SCT was the optimal treatment choice for patients with low/favorable-risk AML, and allo-SCT was the priority selection for patients with intermediate-risk AML, especially young patients. These findings could provide references for clinical practice.

**Supplementary Information:**

The online version contains supplementary material available at 10.1186/s13287-024-03766-5.

## Background

Acute myeloid leukemia (AML) is one of the most common hematological malignancies. Although the development of genetic risk stratification and new treatment strategies has improved outcomes in AML patients in certain subgroups, AML patients still have high mortality [1]. Most AML patients relapse after achieving complete remission (CR) with induction chemotherapy if they do not receive further treatments [2]. The post-remission treatments for patients with AML mainly included consolidated chemotherapy and autologous or allogeneic stem cell transplantation (auto or allo-SCT) [3]. Hematopoietic stem cell transplantation (HSCT) plays an important role in patients with AML [4, 5] and is associated with transplantation-related morbidity and mortality even if it has a high cure rate for AML [6]. The advancement of donor availability and transplantation technology has made allo-SCT the first choice of treatment regime for most adults with high-risk AML because of the high rate of refractory to conventional chemotherapy [7]. Patients with low/favorable-risk AML are usually treated with consolidation chemotherapy in clinical practice even if some studies showed a lower relapse rate with HSCT [8]. The status of HSCT as a post-remission treatment for patients with intermediate-risk AML who achieve CR has remained controversial [9]. There have been many studies comparing the efficacy of post-remission therapies for patients with low/favorable- or intermediate-risk AML, but no consistent conclusion has been formed. Therefore, we conducted this network meta-analysis that combined direct and indirect evidence to compare the curative effects of treatment strategies including allo-SCT, auto-SCT and chemotherapy for patients with low/favorable- or intermediate-risk AML.

## Methods

The study was conducted based on PRISMA statements, and the protocol was registered with CRD42023488606 in PROSPERO.

### Study inclusion and exclusion criteria

Inclusion criteria: patients were diagnosed with low/favorable- or intermediate-risk acute myeloid leukemia (AML); the experimental group was treated with allo-SCT or auto-SCT; the control group was treated with auto-SCT or consolidation chemotherapy (CT); the endpoints of overall survival (OS) and disease-free survival (DFS) were reported in the studies; and studies were clinical trials.

Exclusion criteria: studies of childhood myeloid leukemia; studies of umbilical cord blood stem cell transplantation; studies published repeatedly; studies with incomplete data of results; studies with insufficient follow-up time; studies with more than 20% of patients lost to follow-up.

The two authors independently read the titles and abstracts to screen for studies that may meet the inclusion criteria; subsequently, the two authors independently read the entire texts to select articles that met the inclusion criteria. If there were diverse opinions among the authors, they were resolved through negotiation with a third researcher.

### Search strategy and screening

Study retrieval was conducted with databases including PubMed, Web of Science, Chinese Biomedical Database, Embase and Medline. The search terms and methods were as follows: (1) “stem cell transplantation” or “stem cell transplant”; (2) “acute myeloid leukemia” or “AML”; (3) the first and second terms were merged for retrieval.

### Data extraction

Study information, including the first author, year of publication, age, number of total participants, number of experiment or control group, classification of French-America-British (FAB), risk classification, and endpoints, was collected. All required data from studies were extracted independently by two authors, and if there were diverse opinions among the authors, they could be resolved through negotiation with a third researcher.

### Endpoints of studies

The primary endpoint of the study was OS, and the secondary endpoint was DFS. OS was calculated from the date of using a certain treatment until the date of death (for any cause), and the last follow-up time was usually calculated as the date of death if patients were lost to follow-up before death. DFS was measured from the date of CR until the date of first disease recurrence. The pooled hazard ratios (HRs) and 95% confidence intervals (CIs) for the endpoints were estimated. If the studies did not provide raw data or HRs for endpoints, we used Engauge Digitizer 4.1 software to extract data from the Kaplan-Meier curve and the 1745-625-8-S1 worksheet to calculate HRs and their corresponding 95% CIs.

### Quality assessment

The quality of randomized controlled trials (RCTs) was evaluated with the Cochrane risk-of-bias tool, including randomized methods, blind methods, allocation concealment, incomplete outcome data, selective reporting, and other biases. The cohort studies were evaluated with the Newcastle‒Ottawa quality assessment scale (NOS), which contains three major categories and nine items, including selection (four items), comparability (two items), and exposure or outcome (three items); the scores of studies ranging from 1 to 9 points and with 7–9 points were regarded as high quality.

### Statistical analysis

The pooled HRs and their 95% CIs for the endpoints were estimated with Stata12 and R software. Stata12 software was used to estimate the direct comparison evidence. R software with the JAGS and gemtc packages was used to conduct network meta-analysis, which are based on Bayesian theory and can combine direct and indirect comparisons of evidence. Network meta-analysis can simultaneously compare the differences in treatment effects among multiple interventions and rank them according to the size of the effects [10]. The Node-Splitting method was used to conduct inconsistency test of the network meta-analysis. The pooled HRs of the experimental group versus the control group for endpoints were less than 1, and their 95% CIs did not overlap 1, which indicated that the treatment effect of the experimental group was better. The heterogeneity was calculated with the chi-square test, and there was significant heterogeneity among studies when p was less than 0.05 and I^2^ was greater than 50%. The pooled HRs and their 95% CIs for endpoints were calculated with the random-effects model when significant heterogeneity existed among studies; otherwise, the fixed-effects model was used. Subgroup analysis was adopted to identify the source of heterogeneity.

### Publication biases

We adopted funnel plots and Begg’s and Egger’s tests to estimate the potential publication biases of the included studies. When the funnel plot was symmetrically inverted and funnel-shaped, there was no obvious publication bias. Publication bias was considered to exist when P was less than 0.05.

## Results

### Study identification and selection

**Fig. 1:**
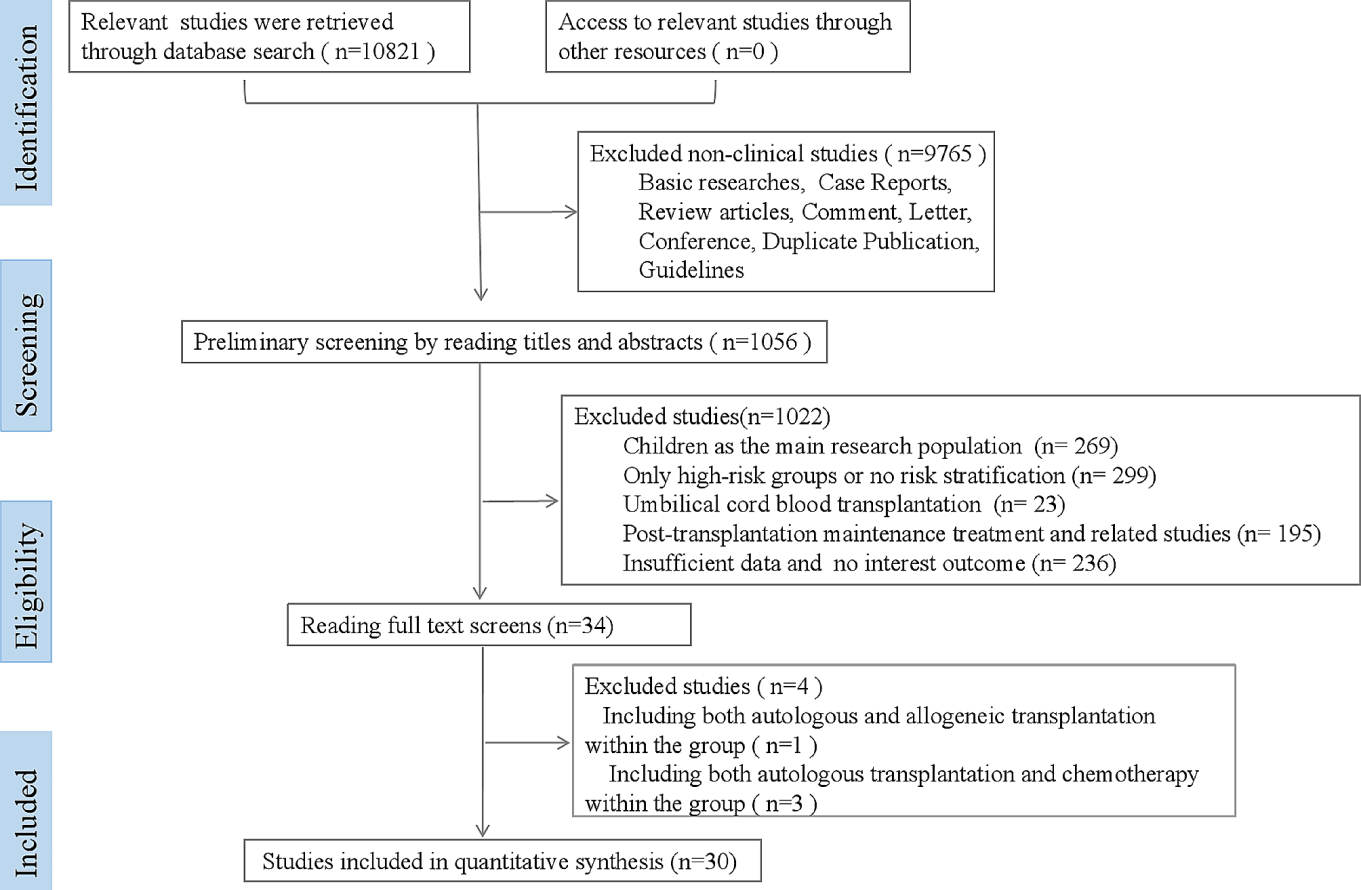
Flow diagram of study screening

A total of 10,821 studies were retrieved initially, and 1056 studies remained when nonclinical studies were excluded, such as basic studies, review articles, case reports and letters. After reading the titles, abstracts, and full texts, there were remaining 34 studies when 1022 studies concerning children as the main research population, only high-risk groups or no risk stratification, umbilical cord blood transplantation, post-transplantation maintenance treatment, insufficient data and no interesting outcomes were excluded. After careful reading of the entire texts, 30 studies were included in the meta-analysis. The screening process of the included studies was performed with a flow chart (Fig. 1).

### Characteristics of the included studies

Fourteen cohort studies and 16 randomized controlled trials [11–34], and 6682 patients were included [9, 35–38]. The characteristics of the studies, including the first author, publication year, age, median follow-up time, treatments and etc. were shown in Table 1. Risk classifications for patients with AML of the included studies were shown in Supplementary Table 1.

**Table 1:** Characteristics of the included studies

First author	Year	Trials’ name	Patients (*n*)	Median age, year (range)	Median follow-up, month (range)	FAB type	Experiment group (CRi)	Control group (CRi)	Experiment(*n*)/ Control(*n*)
Marilyn L. Slovak	2000	E3489/S9034	129	39 (16–55)	60 (NA)	M0-M6	Allo-SCT† (CR1)	Auto-SCT (CR1)	66/63
Stefan Suciu	2003	EORTC/GIMEMAAML−10	288	T: 33 (15–45) C: 35 (15–45)	48 (NA)	M0-M7	Allo-SCT† (donor, CR1)	Auto-SCT (no donor, CR1)	111/177
Eric Jourdan	2005	BGMT	299	T: 34 (15–45) C: 33.5 (15–45)	114 (29–222)	M0-M7	Allo-SCT† (donor, CR1)	Auto-SCT (no donor, CR1)	110/189
Markus Pfirrmann	2012	AML96	260	46 (15–60)	36 (NA)	M0-M2, M4-M7	Allo-SCT† (CR)	Auto-SCT (CR)	117/143
Markus Pfirrmann*	2012	AML2003	141	49 (39–55)	36 (NA)	M0-M2 M4-M7	Allo-SCT† (CR)	Auto-SCT (CR)	109/32
Ki-Seong Eom	2015	NA	59	36.0 (16–64)	60.9 (50.0−71.9)	NA	Allo-SCT‡ (CR1)	Auto-SCT (CR1)	30/29
Jia Chen	2018	NA	195	T: 28 (16–42) C: 34 (24–45)	33 (NA)	NA	Haplo-SCT (CR1)	Auto-SCT (CR1)	107/88
Frederic Baron	2020	EORTC/GIMEMA AML−10	497	T: 33 (15–45) C: 35 (15–45)	132 (NA)	M0-M7	Allo-SCT† (donor, CR)	Auto-SCT (no donor, CR)	185/312
Jean-Luc Harousseau	1997	GOELAM	164	36 (15–50)	62 (NA)	M0-M7	Auto-SCT (CR1)	CT	86/78
Apostolia-Maria Tsimberidou	2003	AML8	34	NA (15–50)	55 (33–64)	M0-M2, M4-M6	Auto-SCT (CR1)	CT	19/15
Dimitri A. Breems	2005	HOVON/ SAKK AML4	130	43 (NA)	154 (NA)	M0-M6	Auto-SCT (CR1)	CT	66/64
Edo Vellenga	2011	AML−29 AML−42	447	T: 49 (16–61) C: 47 (16–61)	106 (13–177)	M0-M2 M4-M7	Auto-SCT (CR1)	CT	231/216
Romain Guieze	2012	GOELAMS LAM−2001	31	47 (18–60)	86 (16–118)	M0-M2 M4-M7	Auto-SCT (CR1)	CT	21/10
Kensuke Usuki	2012	NA	1212	T:48 (16–70) C:52 (16–70)	50 (0.2–116 )	M0-M2 M4-M7	Auto-SCT (CR1)	CT	75/1137
Marie-Anne Hospital	2014	ALFA−9801 ALFA−9802 LAM−2001 CBF−2006	67	43 (16–76)	42 (NA)	NA	Auto-SCT (CR2)	CT	18/49
Toshihiro Miyamoto	2017	NA	87	T: 46.5 (18–63) C: 48 (19–64)	T: 55.1 (3.7−147.9) C: 61.1 ( 4.0−142.0)	M1, M2, M4, M5	Auto-SCT (CR1)	CT	42/45
Adriano Venditti	2019	GIMEMA AML1310	117	49 (18–60)	28.8 (NA)	M0-M2 M4-M7	Auto-SCT (CR1)	CT	19/98
Eun‑Ji Choi	2021	NCT01050036	42	40 (19−60)	55.2 (NA)	NA	Auto-SCT (CR1)	CT	29/13
RF Schlenk	2003	AML HD93	73	46 (16–60)	64 (NA)	M0-M2 M4-M7	Allo-SCT† (CR1)	CT	16/57
Hisashi Sakamaki	2010	AML97	165	T: 37 (16–50) C: 36 (15–50)	NA (3.1−105.8)	M0-M2 M4-M7	Allo-SCT† (donor, CR)	CT (no donor)	73/92
Xiao-Jun Huang	2012	NA	122	T: 30 (16–47) C: 47 (15–60)	19 ( 4–63)	M0-M2 M4-M7	Haplo-SCT (CR1)	CT	52/70
Richard F. Schlenk	2013	HOVON /SAKK AMLSG	92 104	44 (16–60)	62 (NA)	M0-M2 M4-M6	Auto-SCT Allo-SCT† (CR1)	CT	20/72 32/72
Hong-Hu Zhu	2013	AML05	69	33 (15–56)	36 (6–83 )	NA	Allo-SCT£ (CR1)	CT	40/29
Matthias Stelljes	2014	AMLCG99	288	T:45 (16–59) C:46 (17–59)	144 (NA)	M0-M2 M4-M7	Allo-SCT‡ (CR1)	CT	144/144
K. Heidrich	2017	AML2003 AML96	246	48 (39–55)	72 (NA)	M0-M2 M4-M7	Allo-SCT‡ (CR)	CT	97/149
Wasithep Limvorapitak	2018	NA	190	NA(18–65)	104.4 (25.2−181.2)	NA	Allo-SCT† Allo-SCT§ Auto-SCT (CR1)	CT	62/80 18/80 30/80
Meng Lv	2018	NA	147	T:30 (16–58) C:44 (21–60)	48.6 (36.1–88.5)	M0-M2 M4-M6	Haplo-SCT (CR1)	CT	78/69
Adam Folta	2019	NA	116	54 (19–72)	NA	M0-M2 M4-M7	Allo-SCT (NA)	CT	74/42
Nigel H. Russell	2021	AML16	728	NA (60–70)	60 (NA)	M0-M2 M4-M7	Allo-SCT‡ (CR)	CT	122/728
Martin Bornhauser	2023	NCT01246752	143	T: 50.5 (19.0–60.0) C: 51.0 (24.0–60.0)	50 (48–66)	M0-M2 M4-M7	Allo-SCT‡ (CR1)	CT	76/67

*Note* Allo-SCT†: HLA-identical sibling donor; Allo-SCT‡: HLA-matched donor; Allo-SCT£: The donor of more than 50% patients in the group was HLA-matched; Allo-SCT§: Non-related donors, including HLA-mismatched and matched; Markus Pfirrmann *:Data sourced from supplementary materials; Jean-Luc Harousseau 1997: The non-high-risk patients accounted for 88%; Dimitri A. Breems 2005: The non-high-risk patients accounted for 92%; Stefan Suciu 2003: The study was divided into the donor group and the non-donor group, but over 70% patients of the donor group underwent allo-SCT, and over 60% patients of the non-donor group underwent auto-SCT; Eric Jourdan 2005: 94% of patients in the donor group underwent allo-SCT, and 62% of patients in the non-donor group received auto-SCT; Hisashi Sakamaki 2010: More than half of patients in the donor group underwent allo-SCT, with over 87% patients having an intermediate-risk classification Frederic Baron 2020: 71% of patients in the donor group underwent allo-SCT, and 53% of patients in the non-donor group received auto-SCT; *Abbreviation* T, treatment group; C, control group; NA, not available; CT, chemotherapy; CR, complete remission; allo-SCT, allogenic stem cell transplantation; auto-SCT, autologous stem cell transplantation; haplo-SCT, haploidentical stem cell transplantation; FAB, French-American-British

### Quality assessment of the included studies

The quality of the RCTs was evaluated with the Cochrane risk-of-bias tool, and the results showed that the RCTs were considered to be of relatively high quality (Supplementary Table 2). The quality of the cohort studies was evaluated with the NOS, and the mean score was 7.64, ranging from 7 to 8 points, indicating that the quality of the included cohort studies was high (Supplementary Table 3).

### Direct comparison of OS

**Fig. 2:**
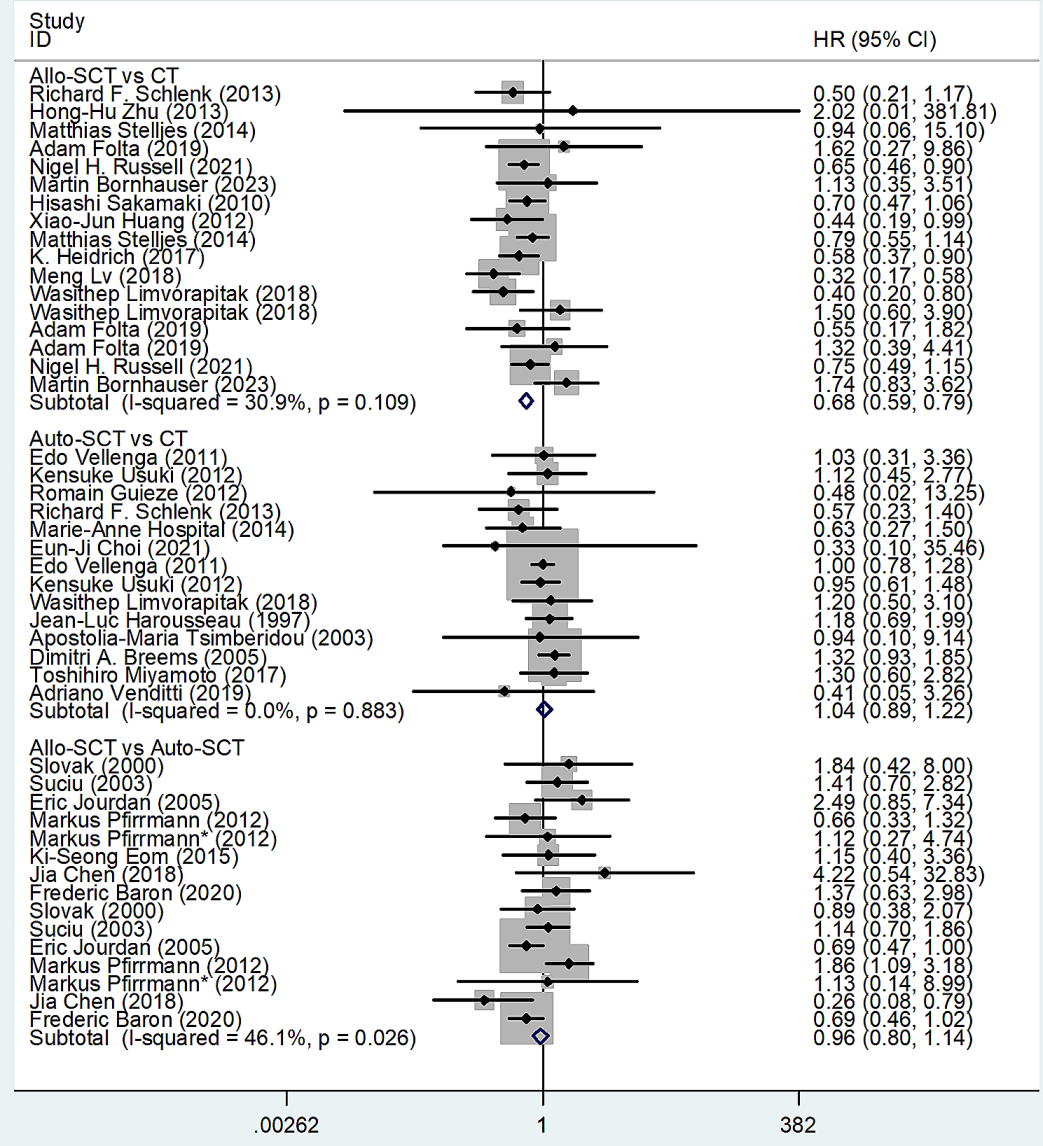
Forest plots of pooled HRs and 95% CIs for OS with direct comparison evaluating the treatment outcomes of allo-SCT, auto-SCT and chemotherapy (CT) in patients with AML. The size of the blocks indicated the weight of the fixed effect model in the meta-analysis

We estimated the pooled HRs and 95% CIs for OS with direct comparison using Stata12 software. The pooled HRs and 95% CIs of the allo-SCT group vs. the CT group, the auto-SCT group vs. the CT group, and the allo-SCT group vs. the auto-SCT group for OS in total patients with AML were 0.68 (95% CI 0.59–0.79), 1.04 (95% CI 0.89–1.22), and 0.96 (95% CI 0.80–1.14), indicating that the OS of the allo-SCT group was longer than that of the CT group, while the OS of the auto-SCT group vs. the CT group and the allo-SCT group vs. the auto-SCT group had no significant difference; the heterogeneity of studies concerning the allo-SCT group vs. the CT group and the auto-SCT group vs. the CT group was not significant, but that of studies concerning the allo-SCT group vs. the auto-SCT group was moderate (Fig. 2).

### Direct comparison of DFS

**Fig. 3:**
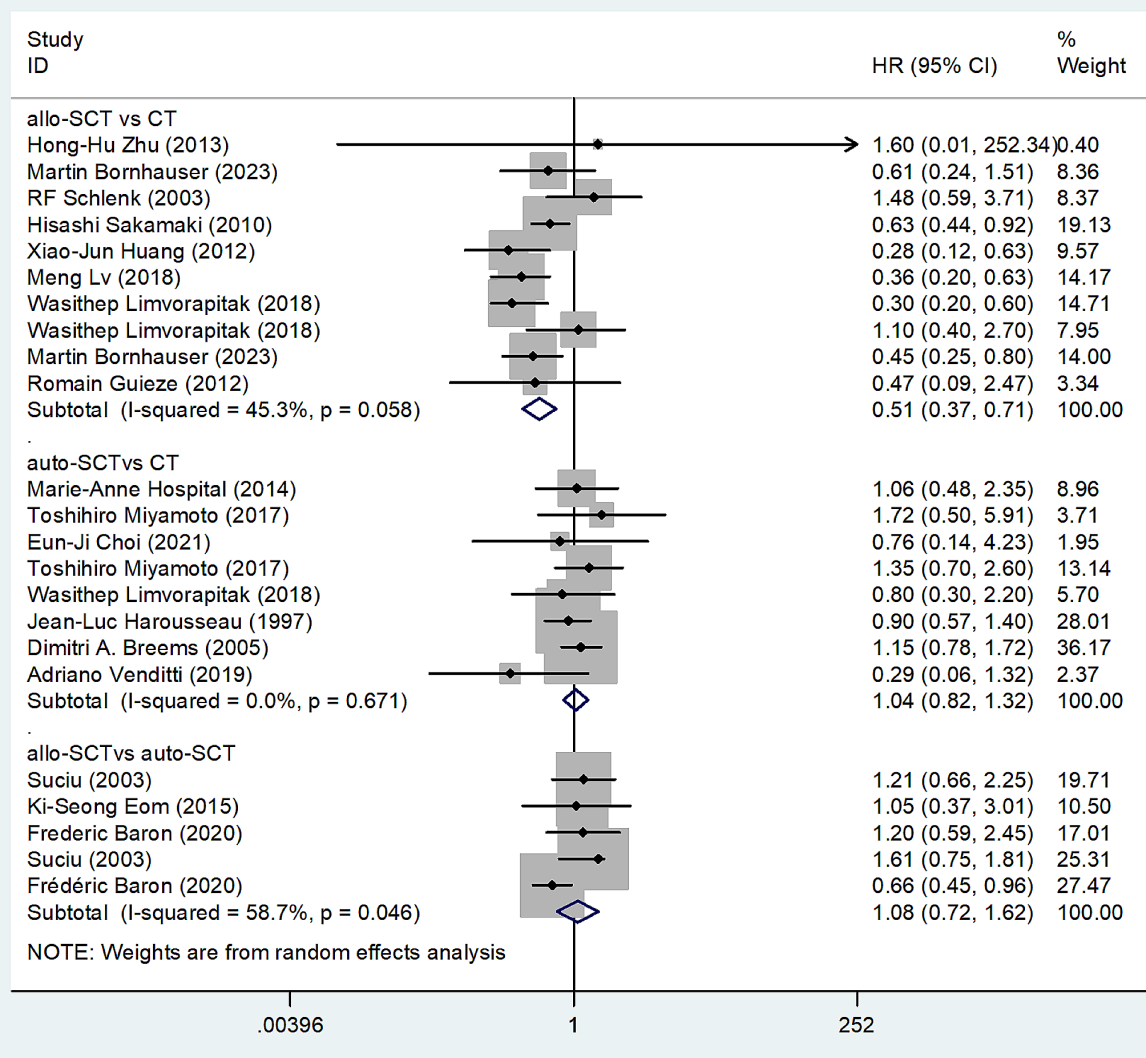
Forest plots of pooled HRs and 95% CIs for DFS with direct comparison evaluating the treatment outcomes of allo-SCT, auto-SCT and chemotherapy (CT) in patients with AML. The size of the blocks indicated the weight of the random effect model in the meta-analysis

We estimated the pooled HRs and 95% CIs for DFS with direct comparison using Stata12 software. The pooled HRs and 95% CIs of the allo-SCT group vs. the CT group, the auto-SCT group vs. the CT group, and the allo-SCT group vs. the auto-SCT group for DFS in total patients with AML were 0.51 (95% CI 0.37–0.71), 1.04 (95% CI 0.82–1.32), and 1.08 (95% CI 0.72–1.62), respectively, indicating that the DFS of the allo-SCT group was better than that of the CT group, while the DFS of the auto-SCT group vs. the CT group and the allo-SCT group vs. the auto-SCT group had no significant difference; the heterogeneity of studies concerning the allo-SCT group vs. the CT group and the auto-SCT group vs. the CT group was not significant, but that of studies concerning the allo-SCT group vs. the auto-SCT group was significant, resulting in the pooled HRs and 95% CIs estimated with a random model (Fig. 3).

### Network-comparison of OS

We estimated the pooled HRs and 95% CIs for OS in AML patients with network-comparison combining direct and indirect evidence.

**Fig. 4:**
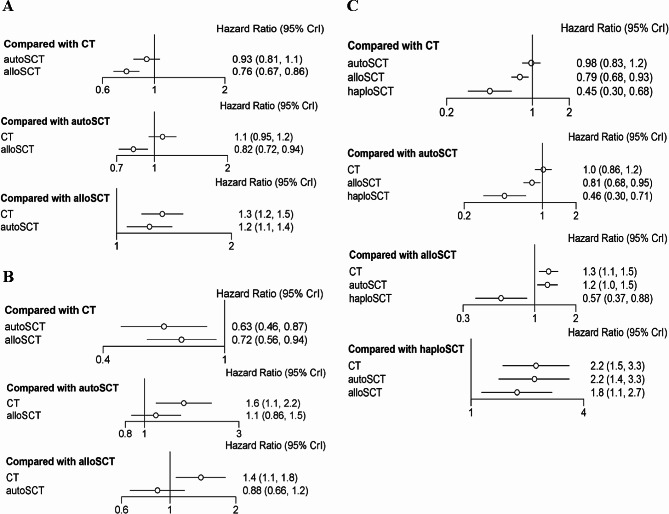
Forest plots of pooled HRs and 95% CIs for OS with network-comparison evaluating the treatment outcomes of allo-SCT including haplo-SCT, auto-SCT and chemotherapy (CT) in patients with AML. (A) in the total patients, (B) in the low/favorable-risk patients, (C) in the intermediate-risk patients

In the total patients with AML, the network plot showed that the comparison of the three groups formed a closed loop (Supplementary Fig. 1A). The median and 97.5% values of the shrink factor tended to be 1 and reached stability after iterative calculation in the convergence diagnostics plot; the number of iterations reached more than 0 in the trace plot, the Markov Chain Monte Carlo (MCMC) chain fluctuated stably and had good overlap; the number of iterations reached 5000 in the density plot, bandwidth tended to be 0 and reached stability; the above results showed that the consistency model of network-meta analysis was a satisfactory convergent model (Supplementary Fig. 1B-C). The OS of the allo-SCT group was longer than that of the auto-SCT group and the CT group, while there was no difference in OS between the auto-SCT group and the CT group (Fig. 4A). The results of rank-probability for OS indicated that the OS of the allo-SCT group was the best, followed by the auto-SCT group, and the CT group was the worst (Table 2).

**Table 2:** Results of rank-probability for OS with preferred direction = -1

	Total	Low risk	Intermediate risk
Allo-SCT	0.001	0.414	0.334
Auto-SCT	0.558	0.092	0.780
Chemotherapy	0.941	0.995	0.865
Haplo-SCT	-	-	0.002

In the patients with low/favorable-risk AML, the network plot showed that the comparison of the three groups formed a closed loop (Supplementary Fig. 2A). The convergence diagnostics, trace, and density plots showed that the consistency model of network meta-analysis was a satisfactory convergent model (Supplementary Fig. 2B-C). The OS of the allo-SCT group and the auto-SCT group was longer than that of the CT group, while there was no difference in OS between the auto-SCT group and the allo-SCT group (Fig. 4B). The results of rank-probability for OS indicated that the OS of the auto-SCT group was the best, followed by the allo-SCT group, and the CT group was the worst (Table 2).

In the patients with intermediate-risk AML, the allo-SCT group excluded haplo-SCT. The network-comparison of the four groups including allo-SCT, haplo-SCT, auto-SCT and CT was shown in Supplementary Fig. 3A. The convergence diagnostics, trace, and density plots showed that the consistency model of network meta-analysis was a satisfactory convergent model (Supplementary Fig. 3B-C). The OS of the allo-SCT group and the haplo-SCT group was longer than that of the auto-SCT group and the CT group, and that of the haplo-SCT group was better than that of the allo-SCT group, while there was no difference in OS between the auto-SCT group and the CT group (Fig. 4C). The results of rank-probability for OS indicated that the OS of the haplo-SCT group was the best, followed by the allo-SCT group, and the auto-SCT group and the CT group were the worst (Table 2).

### Network-comparison of DFS

**Fig. 5:**
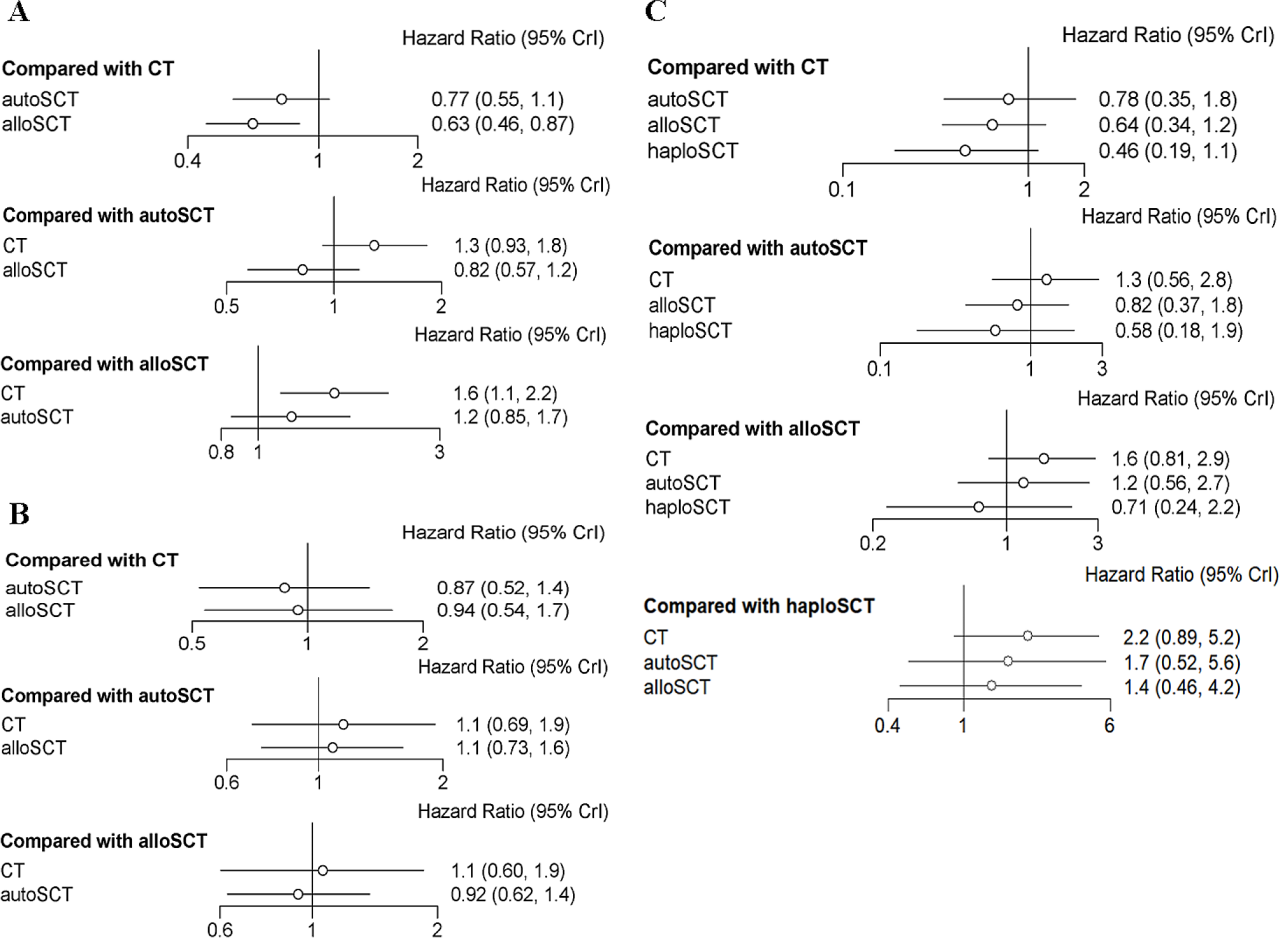
Forest plots of pooled HRs and 95% CIs for DFS with network-comparison evaluating the treatment outcomes of allo-SCT including haplo-SCT, auto-SCT and chemotherapy (CT) in patients with AML. (A) in the total patients, (B) in the low/favorable-risk patients, (C) in the intermediate-risk patients

In the total patients with AML, the network plot showed that the comparison of the three groups formed a closed loop (Supplementary Fig. 4A). The median and 97.5% values of the shrink factor tended to be 1 and reached stability after iterative calculation in the convergence diagnostics plot; the number of iterations reached more than 20,000 in the trace plot, the MCMC chain fluctuated stably and had good overlap; the number of iterations reached 5000 in the density plot, bandwidth tended to be 0 and reached stability; the above results showed that the consistency model of network meta-analysis was a relatively satisfactory convergent model (Supplementary Fig. 4B-C). The DFS of the allo-SCT group was better than that of the CT group, while there was no difference in DFS between the auto-SCT group and the CT group or the allo-SCT group and the auto-SCT group (Fig. 5A). The results of rank-probability for DFS indicated that the DFS of the allo-SCT group was the best, followed by the auto-SCT group, and the CT group was the worst (Table 3).

**Table 3:** Results of rank-probability for DFS with preferred direction = -1

	Total	Low risk	Intermediate risk
Allo-SCT	0.065	0.534	0.369
Auto-SCT	0.468	0.325	0.605
Chemotherapy	0.967	0.641	0.875
Haplo-SCT	-	-	0.151

In the patients with low/favorable-risk AML, the network plot showed that the comparison of the three groups formed a closed loop (Supplementary Fig. 5A). The convergence diagnostics, trace, and density plots showed that the consistency model of network meta-analysis was a satisfactory convergent model (Supplementary Fig. 5B-C). The DFS among the allo-SCT group, the auto-SCT group and the CT group was not different (Fig. 5B). The results of rank-probability for DFS indicated that the DFS of the auto-SCT group was most likely to be ranked first, followed by the allo-SCT group and the CT group (Table 3).

In the patients with intermediate-risk AML, the allo-SCT group excluded haplo-SCT. The network-comparison of the four groups including allo-SCT, haplo-SCT, auto-SCT and CT was shown in Supplementary Fig. 6A. The convergence diagnostics, trace, and density plots showed that the consistency model of network meta-analysis was a relatively satisfactory convergent model (Supplementary Fig. 6B-C). The DFS among the allo-SCT group, the haplo-SCT group, the auto-SCT group and the CT group was not different (Fig. 5C). The results of rank-probability for DFS indicated that the DFS of the haplo-SCT group was most likely to be ranked first, followed by the allo-SCT group, and the auto-SCT group and the CT group were likely ranked last (Table 3).

### Inconsistency and heterogeneity tests

We conducted an inconsistency test of the network meta-analysis with the Node-Splitting method. The inconsistency test of OS and DFS in total patients with AML indicated that the direct, indirect and network comparisons of the allo-SCT group vs. the CT group, the auto-SCT group vs. the CT group, and the allo-SCT group vs. the auto-SCT group could not meet the conditions of the consistency check. However, in the subgroups according to risk classification, the direct, indirect and network comparisons among groups satisfied the consistency test with *P* > 0.05 (Supplementary Figs. 7–8).

The global I-squared in heterogeneity test for OS indicated that there was no significant heterogeneity in the total patients or subgroups (Supplementary Table 4a-c). The global I-squared in heterogeneity test for DFS indicated that there was no significant heterogeneity in patients with low-risk AML, while there was significant heterogeneity in total patients or in patients with intermediate-risk AML (Supplementary Table 4d-f).

### Publication bias

**Fig. 6:**
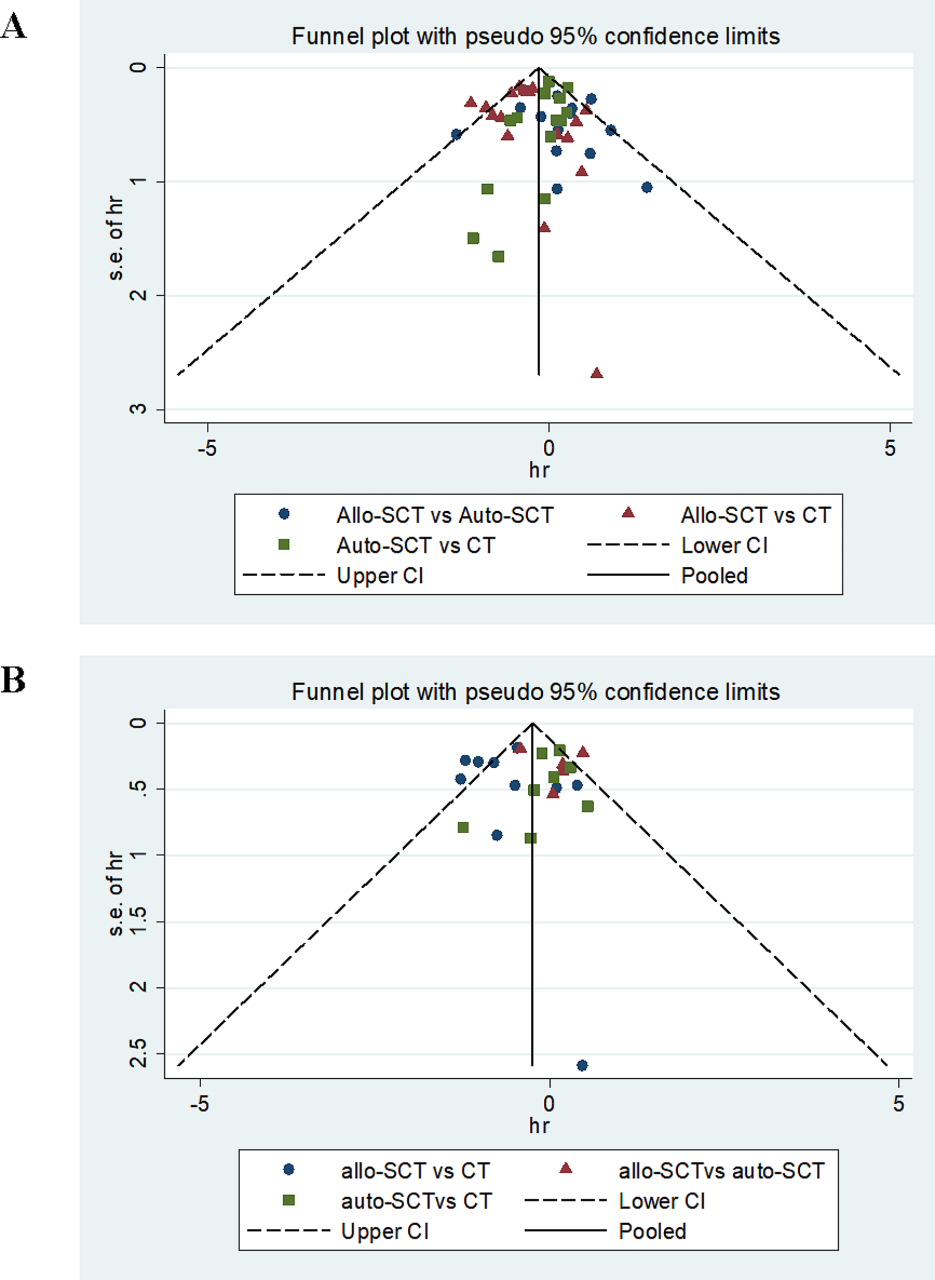
Funnel plots of the included studies for OS (**A**) and DFS (**B**)

The funnel plots of the included studies for OS and DFS were symmetrically inverted and funnel-shaped, indicating that there was no obvious publication bias (Fig. 6). Begg’s test (*P* = 0.722) and Egger’s test (*P* = 0.715) of the included studies for OS showed that no publication bias existed (Supplementary Fig. 9). Begg’s test (*P* = 0.781) and Egger’s test (*P* = 0.632) of the included studies for DFS showed that no publication bias existed (Supplementary Fig. 10).

## Discussion

AML is a phenotypic and prognostic heterogeneous hematopoietic stem cell disease [39]. In recent years, the outcomes of AML patients have been continuously improved with the development of drug therapy, but HSCT is still an indispensable treatment with curative potential for patients [40, 41]. The therapeutic status of allo-SCT as a post-remission treatment for patients with high-risk AML is relatively recognized; however, the optimal treatment for patients with low/favorable- or intermediate-risk AML who achieve CR has remained controversial. Therefore, we conducted this network meta-analysis to discuss this disputed problem.

In the total AML patients, the OS of the allo-SCT group was longer than that of the auto-SCT group and the CT group, while there was no difference in OS between the auto-SCT group and the CT group. The results of rank-probability for OS indicated that the OS of the allo-SCT group was the best, followed by the auto-SCT group, and the CT group was likely to be the worst. The DFS of the allo-SCT group was better than that of the CT group, while there was no difference in DFS between the auto-SCT group and the CT group or the allo-SCT group and the auto-SCT group. The results of rank-probability for DFS indicated that the DFS of the allo-SCT group was likely to be the best, followed by the auto-SCT group, and the CT group may be the worst. In the patients with low/favorable-risk AML, the OS of the allo-SCT group and the auto-SCT group was longer than that of the CT group, while there was no difference in OS between the auto-SCT group and the allo-SCT group. The results of rank-probability for OS indicated that the OS of the auto-SCT group was likely to be the best, followed by the allo-SCT group, and the CT group was the worst. The DFS among the allo-SCT group, the auto-SCT group and the CT group was not different. The results of rank-probability for DFS indicated that the DFS of the auto-SCT group was most likely to be ranked first, followed by the allo-SCT group and the CT group. In the patients with intermediate-risk AML, the allo-SCT group excluded haplo-SCT. The OS of the allo-SCT group and the haplo-SCT group was longer than that of the auto-SCT group and the CT group, that of the haplo-SCT group was better than that of the allo-SCT group, while there was no difference in OS between the auto-SCT group and the CT group. The results of rank-probability for OS indicated that the OS of the haplo-SCT group was the best, followed by the allo-SCT group, and the auto-SCT group and the CT group were the worst. The DFS among the allo-SCT group, the haplo-SCT group, the auto-SCT group and the CT group was not different. The results of rank-probability for DFS indicated that the DFS of the haplo-SCT group was most likely to be ranked first, followed by the allo-SCT group, and the auto-SCT group and the CT group were likely ranked last. However, the median age of the haplo-SCT group was much younger than that of the control group in the included studies concerning haplo-SCT, which may be one of the reasons for the better prognosis of the haplo-SCT group. Therefore, the conclusion that haplo-SCT was the best treatment in intermediate-risk AML patients remained unreliable.

Our study was the first network meta-analysis to discuss the controversial problem of which was the most optimal treatment among allo-SCT, auto-SCT and CT for patients with low/favorable- or intermediate-risk AML. This article included a relatively large number of studies to compare three interventions, and there was no significant publication bias in the included studies. Network meta-analysis integrated indirect and direct evidence, which could improve the efficiency of statistical analysis and form more reliable conclusions than individual studies [42, 43]. However, there were several limitations in our study. First, the pooled effects were estimated from the extracted data of RCTs and cohort studies instead of from the raw data. Second, we did not estimate other endpoints, such as treatment-related mortality or cumulative incidence of relapse, due to the limited number of related studies.

## Conclusions

The group of low/favorable- and intermediate-risk patients was the total population that was discussed in the meta-analysis. The part of clinical studies included in our meta-analysis did not conduct subgroup analysis based on risk stratification in the low/favorable- and intermediate-risk AML patients, which means that the study population of these clinical studies was non-high-risk AML patients. Therefore, we first conducted a meta-analysis in the total population and found that these patients should prioritize allo-SCT if they are eligible for transplantation, and auto-SCT is optional. However, in the subgroup analysis of the included studies that conducted subgroup analysis based on risk stratification, the results indicated that auto-SCT was the optimal treatment choice for patients with low/favorable-risk AML, and allo-SCT was the priority selection for patients with intermediate-risk AML, especially young patients. These findings could provide references for clinical practice.

### Electronic supplementary material

Below is the link to the electronic supplementary material.

Supplementary Material 1

## Data Availability

All data relevant to the study are included in the article or uploaded as supplementary information.

## Abbreviations

AML: Acute myeloid leukemia
CR: Complete remission
HSCT: Hematopoietic stem cell transplantation
auto-SCT: Autologous stem cell transplantation
allo-SCT: Allogeneic stem cell transplantation
CT: Chemotherapy
OS: Overall survival
DFS: Disease-free survival
FAB: French-America-British
HRs: Hazard ratios
CIs: Confidence intervals
RCTs: Randomized controlled trials
