# Supplementary material for: Comparison of allo-SCT, auto-SCT and chemotherapy for the treatment of patients with low- or intermediate-risk acute myeloid leukemia: a network meta-analysis
Source: Stem Cell Res Ther. 2024 May 31;15:153. doi: 10.1186/s13287-024-03766-5 (PMC11141018; doi:10.1186/s13287-024-03766-5)
Supplement: Supplementary file 1 — Supplementary Material 1 [file 13287_2024_3766_MOESM1_ESM.pdf]

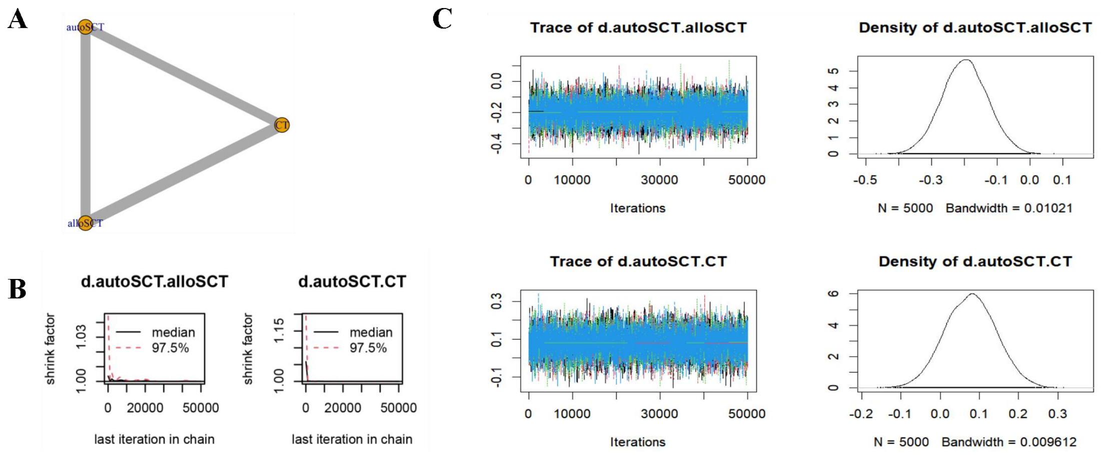


**Supplementary Figure 1** The network plot (A), convergence diagnostics plot (B), and trace and density plot (C) for OS in the total AML patients with network meta-analysis

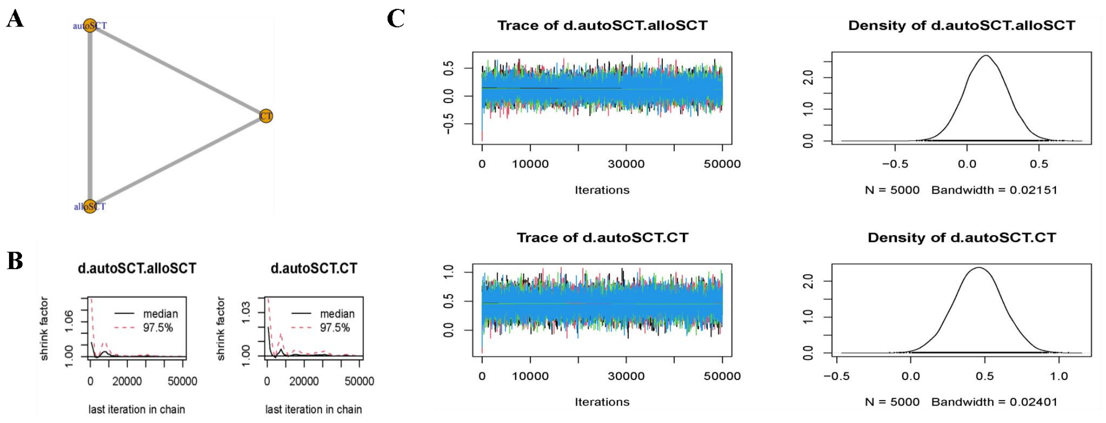


**Supplementary Figure 2** The network plot (A), convergence diagnostics plot (B), and trace and density plot (C) for OS in the low/favorable-risk AML patients with network meta-analysis

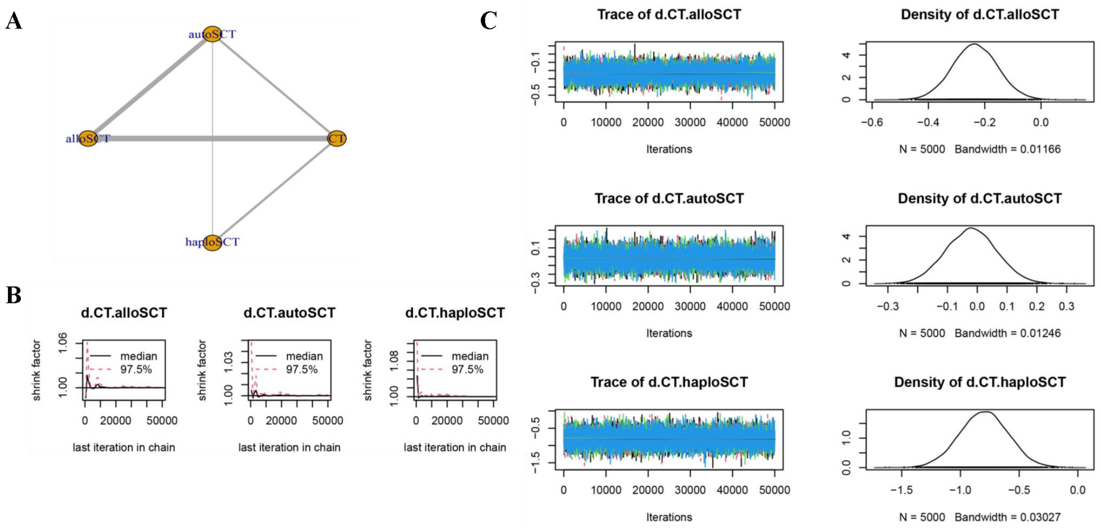


**Supplementary Figure 3** The network plot (A), convergence diagnostics plot (B), and trace and density plot (C) for OS in the intermediate-risk AML patients with network meta-analysis

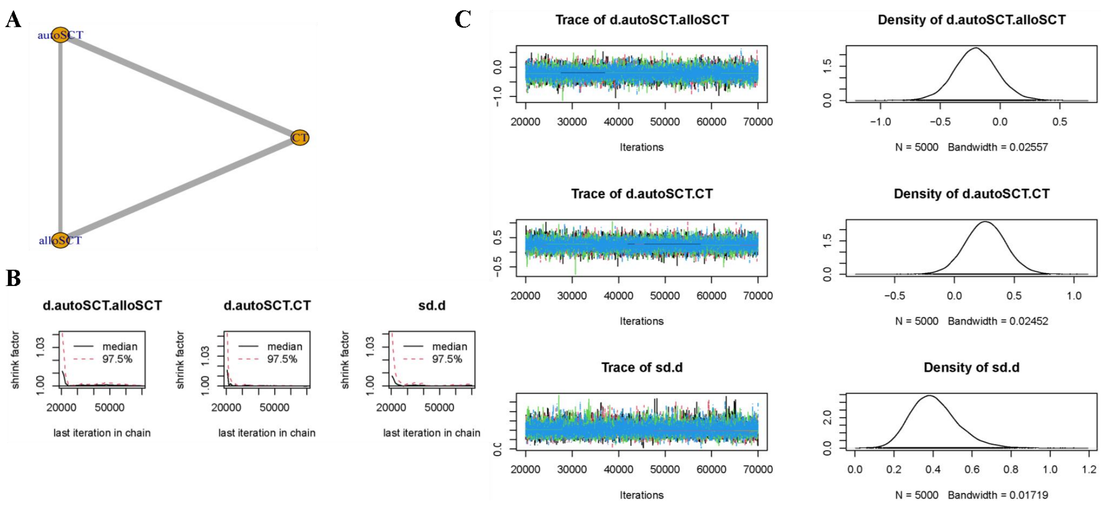


**Supplementary Figure 4** The network plot (A), convergence diagnostics plot (B), and trace and density plot (C) for DFS in the total AML patients with network meta-analysis

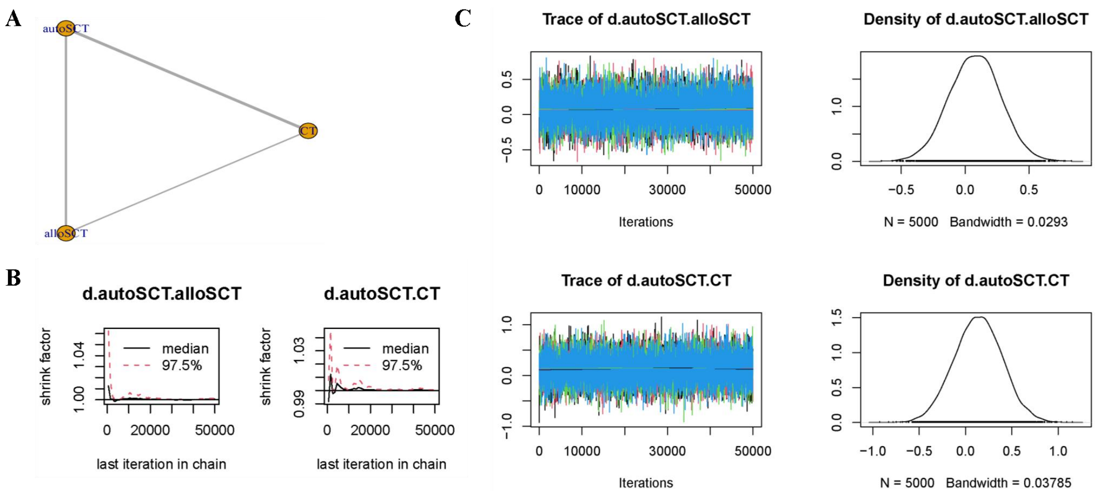


**Supplementary Figure 5** The network plot (A), convergence diagnostics plot (B), and trace and density plot (C) for DFS in the low/favorable-risk AML patients with network meta-analysis

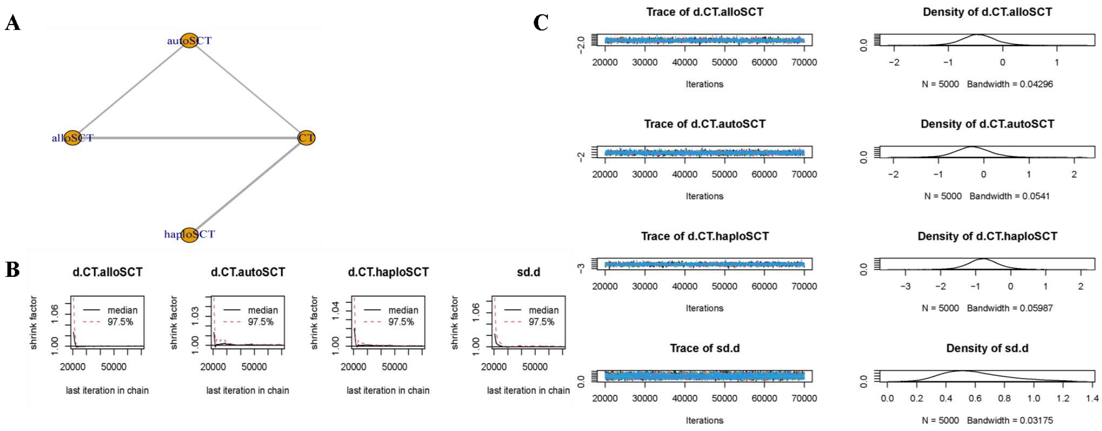


**Supplementary Figure 6** The network plot (A), convergence diagnostics plot (B), and trace and density plot (C) for DFS in the intermediate-risk AML patients with network meta-analysis

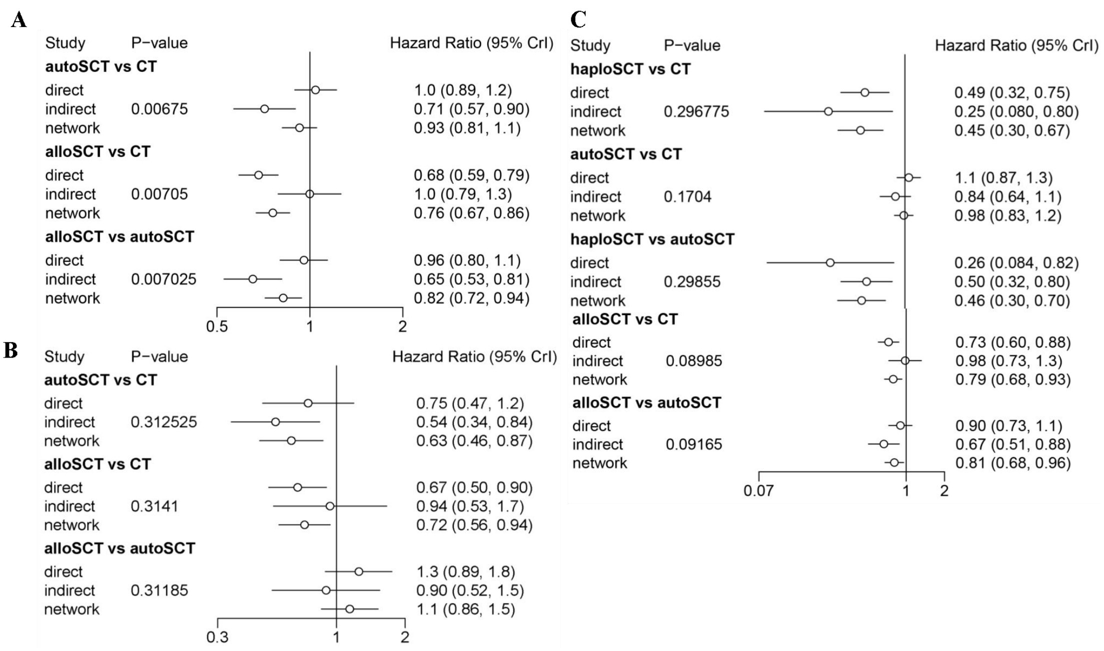


**Supplementary Figure 7** The inconsistency detection of direct, indirect and network comparisons for OS in AML patients with the Node-Splitting method. A. in the total patients, B. in the low/favorable-risk patients, C. in the intermediate-risk patients

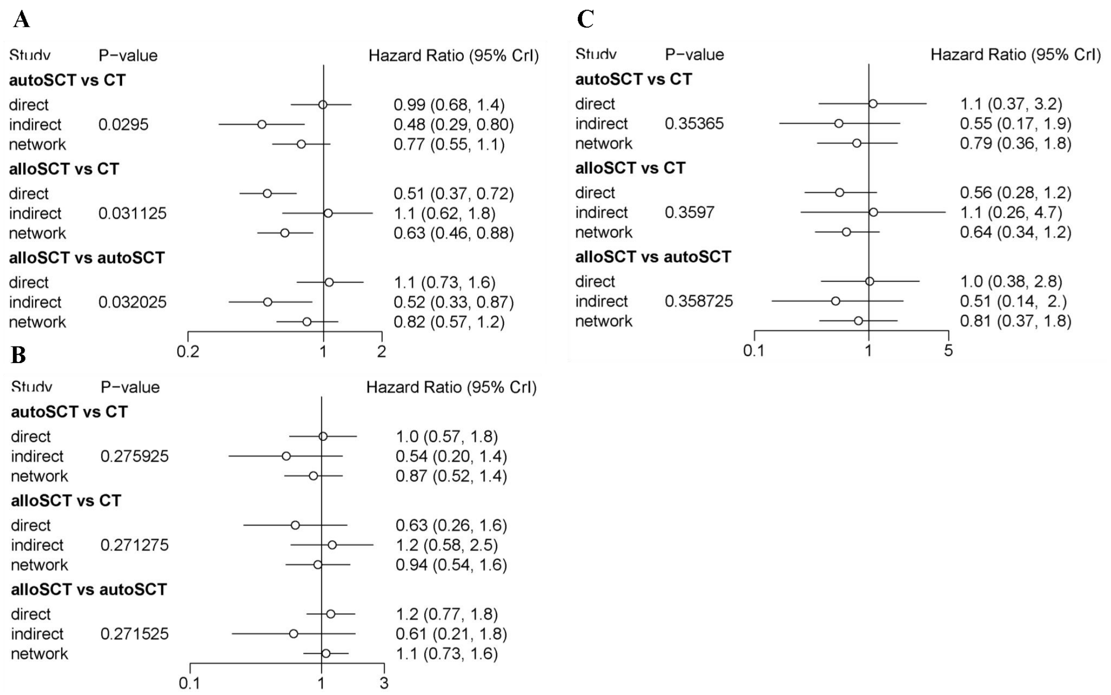


**Supplementary Figure 8** The inconsistency detection of direct, indirect and network comparisons for DFS in AML patients with the Node-Splitting method. A. in the total patients, B. in the low/favorable-risk patients, C. in the intermediate-risk patients

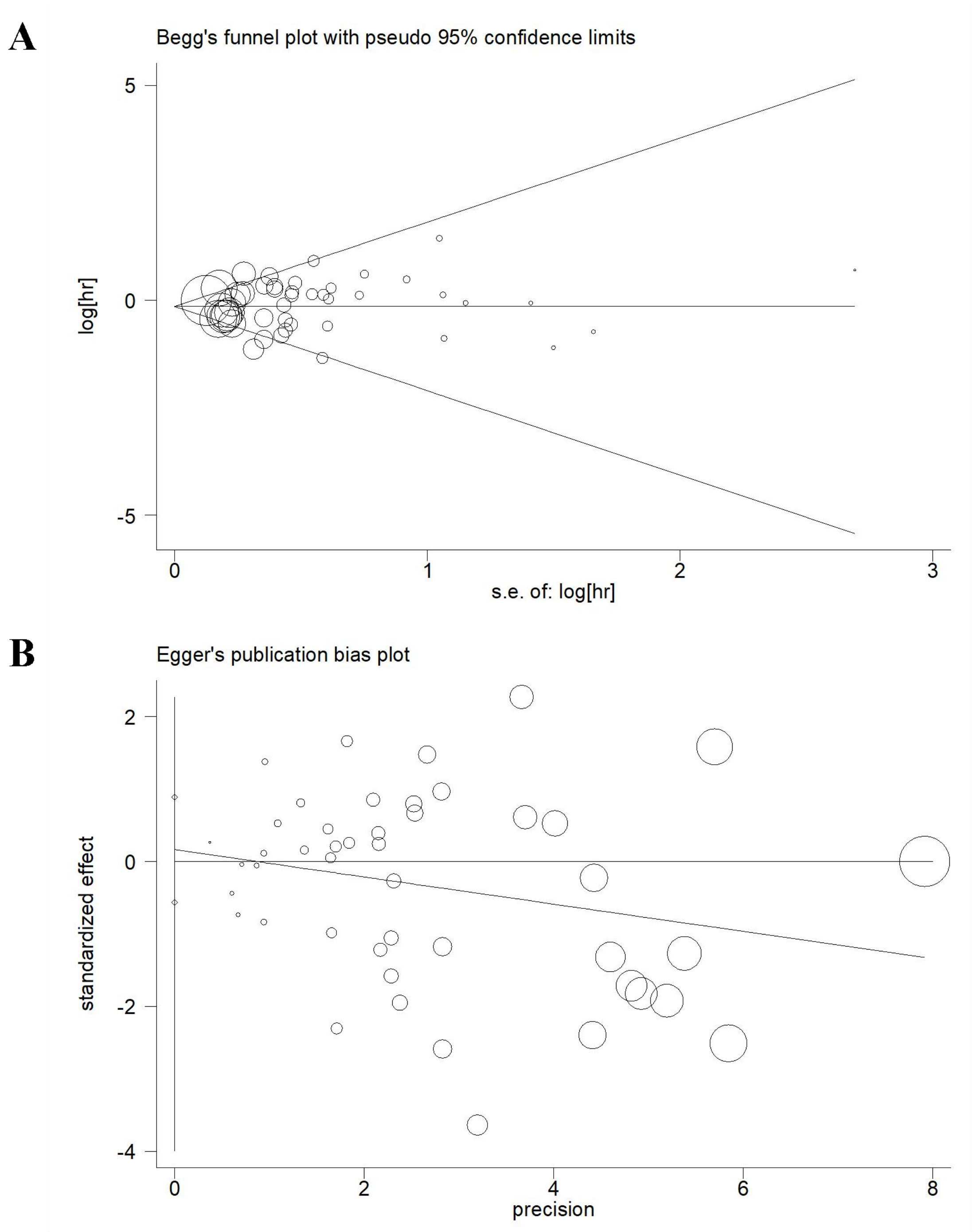


**Supplementary Figure 9** Begg's test (A) with P=0.722 and Egger's test (B) with P=0.715 of the included studies for OS

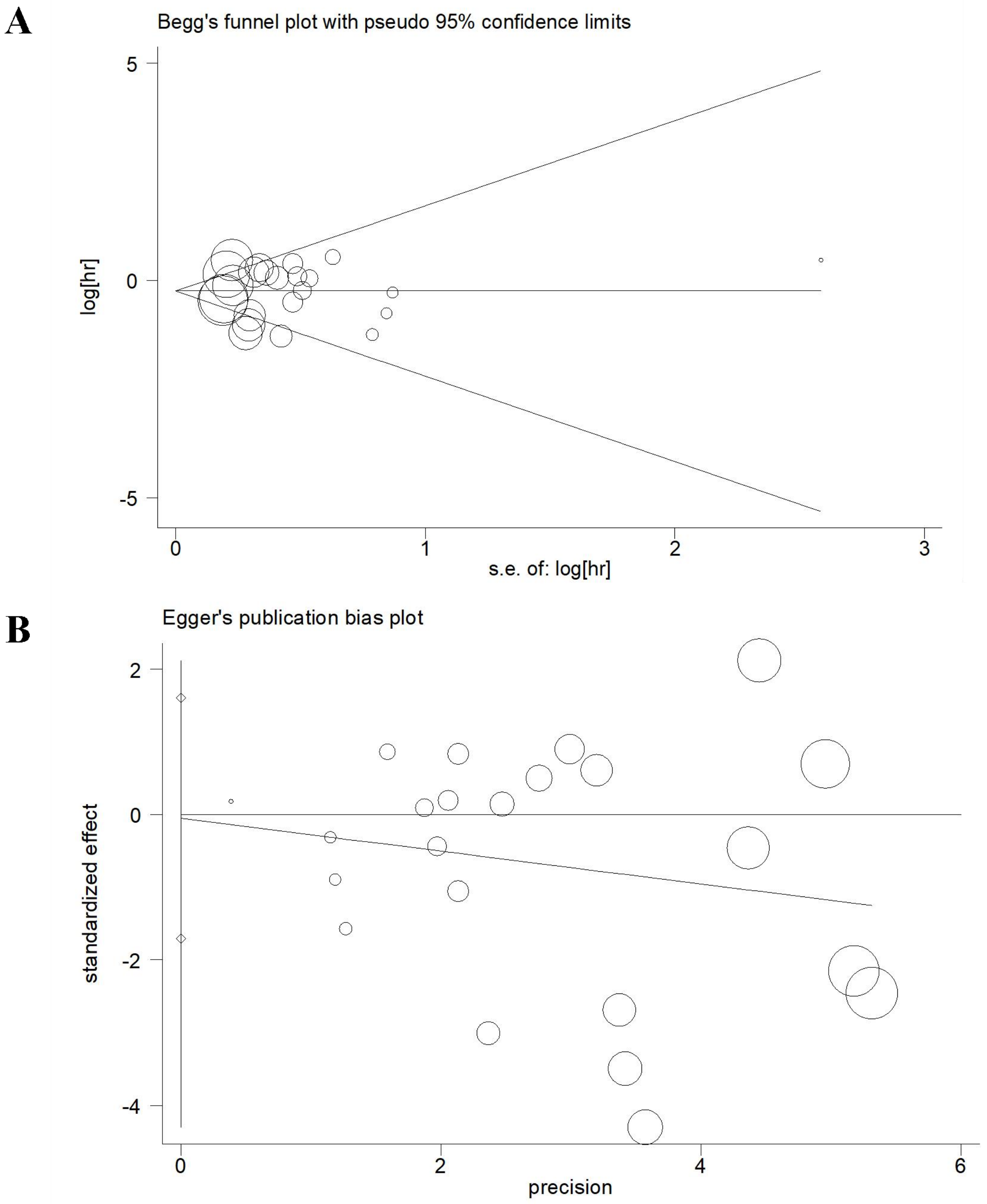


**Supplementary Figure 10** Begg’s test (A) with P=0.781 and Egger’s test (B) with P=0.632 of the included studies for DFS

**Supplementary Table 1** Risk stratification of the included studies

| First author | Year | Risk stratification |
|---|---|---|
| Marilyn L. | 2000 | Low risk: inv(16)/t(16;16)/del(16q), t(15;17) with/without secondary aberrations; t(8;21) lacking del(9q) or complex karyotypes |
| Slovak | | Intermediate risk: normal, 18, 16, 2Y, del(12p) |
| Stefan | 2003 | Low risk: t(8;21) or inv(16) |
| Suciu | | Intermediate risk: NN or -Y only |
| Eric | 2005 | Low risk: t(15;17), t(8;21), or inv(16) |
| Jourdan | | Intermediate risk: presence of other abnormalities without low- or high-risk cytogenetics which were presented of -5, 5q- , -7, abn 3q, t(9;22), t(6;9), abn 11q23 except t(9;11) complex karyotypes (≥three abnormalities) |
| Markus | 2012 | Favorable risk: post-remission treatment scores |
| Pfirrmann | | Intermediate risk: post-remission treatment scores |
| Markus | 2012 | Favorable risk: post-remission treatment scores |
| Pfirrmann∗ | | Intermediate risk: post-remission treatment scores |
| Ki-Seong | 2015 | t(8;21) |
| Eom | | |
| Jia | 2018 | Favorable risk: NCCN 2017, version 1 |
| Chen | | Intermediate risk: NCCN 2017, version 1 |
| Frederic | 2020 | Favorable risk: t(15;17)(q22;q21), t(8;21)(q22;q22), and inv(16)(p13q22)/t(16;16)(p13;q22) |
| Baron | | Intermediate risk: presence of other abnormalities without favorable- or high-risk cytogenetics; high-risk cytogenetics were presented of abn(3q) (excluding t(3;5)(q25;q34)), inv(3)(q21q26)/t(3;3)(q21;q26), add(5q)/del(5q), -5, -7, add(7q)/del(7q), t(6;11)(q27;q23), t(10;11)(p11 approximately 13;q23), other t(11q23) (excluding t(9;11)(p21 approximately 22;q23) and t(11;19)(q23;p13)), t(9;22)(q34;q11), -17, and abn(17p) |
| Jean-Luc | 1997 | Low risk: t(8;21) ,t(15;17) or inv(16) |
| Harousseau | | Intermediate risk: presence of other abnormalities without unfavorable (−5, 5q−, −7, or multiple abnormalities) and low risk |
| Apostolia-Maria | 2003 | Low risk: t(8;21) or inv(16) |
| Tsimberidou | | Intermediate risk: normal karyotype (+8 or less than three numerical abnormalities), excluding those involving chromosomes 5 or 7 |
| Dimitri A. | 2005 | Favorable risk: favorable risk was defined by the presence of t(8;21) and a white blood cell count of $<20*10^9$/l at diagnosis without additional unfavourable |

| | | |
|---|---|---|
| Breems | | cytogenetic abnormalities or the presence of t(15;17), t(16;16) or inv(16)(p13;q22) without additional unfavourable cytogenetic abnormalities<br>Intermediate risk: the intermediate risk group contained patients who did not meet the criteria for favourable or unfavourable risk; unfavourable risk was presented of -5, -7, del 5q-, del 7q-, abn 3q, t(6;9), t(9;22), abn 11q23 and complex cytogenetic abnormalities |
| Edo Vellenga | 2011 | Favorable risk: core-binding factor abnormalities: t(8;21) (q22;q22), inv(16)(p13.1;q22), or t(16;16)(p13.1;q22)<br>Intermediate risk: normal karyotype and presence of other abnormalities without unfavorable and low risk; unfavorable risk was presented with a monosomal karyotype, complex abnormalities, t(6;9), t(11,19), t(9;22), 11q23.3q, inv(3),-5q ,-7q, -5, or -7 |
| Romain Guieze | 2012 | NPM1 (NPM1+) without FLT3-ITD, and normal karyotype |
| Kensuke Usuki | 2012 | Favorable risk: inv(16)/t(16;16)/del(16q), t(15;17) with/without secondary aberrations; t(8;21) lacking del(9q) or complex karyotypes<br>Intermediate risk: normal, +8, +6, −Y, del(12p) |
| Marie-Anne Hospital | 2014 | t(8;21), inv(16)/t(16;16) |
| Toshihiro Miyamoto | 2017 | Favorable risk: t(8;21)/AML1-MTG8, inv(16) or t(16;16)/CBFb-MYH11<br>Intermediate risk: normal karyotype and presence of other abnormalities without unfavorable and low risk; unfavorable-risk exhibited monosomal karyotype, t(6;9)/DEK-CAN, t(9;22)/BCR-ABL, t(6;11)/MLL-AF6, t(9;11)/MLL-AF9, or t(11;19)/MLL-ENL |
| Adriano Venditti | 2019 | Favorable risk: NCCN 2009, version 1<br>Intermediate risk:NCCN 2009, version 1 |
| Eun-Ji Choi | 2021 | t(8;21) or inv(16)/t(16;16) |
| RF Schlenk | 2003 | Intermediate risk: normal karyotype, and excluding low risk which exhibited t(8;21) or inv/t(16q22) irrespective of additional chromosomal aberrations or high risk which included all other chromosomal abnormalities |
| Hisashi Sakamaki | 2010 | Intermediate risk: JALSG scoring system (4-6 points) |
| Xiao-Jun Huang | 2012 | Intermediate risk: patients without favorable or adverse abnormalities; cytogenetic abnormalities t(8;21)(q22;q22), t(15;17)(q22;q21), and inv(16) or t(16;16)(p13;q22) were considered as favorable risk; complex cytogenetic abnormalities and -5/5q- , -7/7q- , abn(3q), t(6;9)(q23;q34), abn(11q23), and t(9;22)(q34;q11) were considered as high risk |

| | | |
|---|---|---|
| Richard F. Schlenk | 2013 | Favorable risk: double mutant CEBPA |
| Hong-Hu Zhu | 2013 | t(8;21) |
| Matthias Stelljes | 2014 | Favorable risk: t(8;21); inv(16) or t(16;16); normal karyotype (NK) with mutated NPM1 and no FLT3 ITD; NK with double-mutated CEBPA<br>Intermediate risk: NK with wild-type NPM1 and no FLT3-ITD; presence of other abnormalities without unfavorable and favorable risk; unfavorable karyotype was defined as complex karyotype, −5/5q-, −7/7q-, abnormal 3q21/3q26, or abnormal 11q23 |
| K. Heidrich | 2017 | Intermediate risk AML without FLT3-ITD, biallelic CEBPA-, or NPM1 mutations |
| Wasithep Limvorapitak | 2018 | Intermediate risk: presence of other abnormalities without unfavorable and favourable risk; favorable risk included t(15;17), t(8;21) and inv(16)/t(16;16) irrespective of additional cytogenetics abnormalities; unfavorable karyotype included abnormal 3q (including t(3;5)), inv(3)/t(3;3), add(5q), del(5q), -5, -7, add(7q)/del(7q), t(6;11), t(10;11), t(11q23) (excluding t(9;11) and t(11;19)), t(9;22), -17/abn(17p), and 4 or more complex unrelated abnormalities |
| Meng Lv | 2018 | Intermediate risk: excluding favorable abnormalities t(8;21)(q22;q22), t(15;17)(q22;q21), inv(16) or t(16;16)(p13;q22), and NPM1+FLT3-ITD-; excluding adverse risk abnormalities, complex cytogenetic abnormalities (defined as at least three unrelated cytogenetic clones), monosomal karyotype, -5/5q-, -7/7q-, inv(3)(q21.3q26.2)/t(3;3)(q21.3;q26.2), t(6;9)(q23;q34), abn(11q23)-none t(9;11), and t(9;22)(q34;q11) |
| Adam Folta | 2019 | Favorable risk: DNMT3A-/RCS-/(NPM1+ or CEBPA+)<br>Intermediate risk: DNMT3A-/RCS-/NPM1-/ CEBPA- or DNMT3A-/FLT3-ITD-/RCS- |
| Nigel H. Russell | 2021 | Favorable risk: t(8;21), inv(16), irrespective of the presence of other abnormalities<br>Intermediate risk: normal karyotype and presence of other abnormalities without adverse and favorable risk; adverse risk was presented with monosomy 5, monosomy 7, del(5q), abnormal 3q, and complex (5 or more chromosomal abnormalities) |
| Martin Bornhauser | 2023 | Favorable risk: t(8;21)(q22;q22.1); RUNX1-RUNX1T1; inv(16)(p13.1q22) or t(16;16)(p13.1;q22); CBFB-MYH11; mutated NPM1 without FLT3-ITD or with FLT3-ITD $^{low}$; biallelic mutated CEBPA (2017 ELN risk stratification)<br>Intermediate risk: mutated NPM1 and FLT3-ITD$^{high}$; wild-type NPM1 without FLT3-ITD or with FLT3-ITD$^{low}$ (without adverse-risk genetic lesions); t(9;11)(p21.3;q23.3); MLLT3-KMT2A; cytogenetic abnormalities not classified as favorable or adverse (2017 ELN risk stratification) |

**Abbreviation:**

NCCN: National Comprehensive Cancer Network

ELN: European Leukemia Network

**Supplementary Table 2** Quality evaluation of the included studies with Cochrane risk-of-bias tool

| First author | Year | Randomized methods | Blind methods | Allocation concealment | Incomplete outcome data | Selective reporting | Other biases |
|---|---|---|---|---|---|---|---|
| Stefan Suciu | 2003 | Low risk | Unclear | Unclear | Low risk | Low risk | High risk |
| Eric Jourdan | 2005 | Low risk | Unclear | Unclear | Low risk | Low risk | High risk |
| Markus Pfirrmann | 2012 | Low risk | Unclear | Unclear | Low risk | Low risk | Unclear |
| Markus Pfirrmann∗ | 2012 | Low risk | Unclear | Unclear | Low risk | Low risk | Unclear |
| Frederic Baron | 2020 | Low risk | Unclear | Unclear | Low risk | Low risk | High risk |
| Jean-Luc Harousseau | 1997 | Low risk | Unclear | Unclear | Low risk | Low risk | Unclear |
| Apostolia-Maria Tsimberidou | 2003 | Low risk | Unclear | Unclear | Low risk | Low risk | High risk |
| Dimitri A. Breems | 2005 | Low risk | Unclear | Unclear | Low risk | Low risk | High risk |
| Edo Vellenga | 2011 | Low risk | Unclear | Unclear | Low risk | Low risk | Unclear |
| Romain Guieze | 2012 | Low risk | Unclear | Unclear | Low risk | Low risk | High risk |
| Toshihiro Miyamoto | 2017 | Low risk | Unclear | Unclear | Low risk | Low risk | Unclear |
| RF Schlenk | 2003 | Low risk | Unclear | Unclear | Low risk | Low risk | High risk |
| Hisashi Sakamaki | 2010 | Low risk | Unclear | Unclear | Low risk | Low risk | High risk |
| Matthias Stelljes | 2014 | Low risk | Unclear | Unclear | Low risk | Low risk | Unclear |
| K. Heidrich | 2017 | Low risk | Unclear | Unclear | Low risk | Low risk | Unclear |
| Martin Bornhauser | 2023 | Low risk | Unclear | Unclear | Low risk | Low risk | Unclear |

**Note:**

Markus Pfirrmann∗: Data sourced from supplementary materials

**Supplementary Table 3** Assessment of methodological quality of the included studies with the Newcastle-Ottawa Scale

| First author | Year | Selection | | | | Comparability | Outcome | | | Score |
|---|---|---|---|---|---|---|---|---|---|---|
| | | Representativeness of exposed cohort | Selection of non exposed cohort | Ascertainment of exposure | Outcome of interest not presented at start | | Assessment of outcome | Follow-up length | Follow-up adequacy | |
| Ki-Seong Eom | 2015 | * | * | * | * | * | * | * | * | 8 |
| Jia Chen | 2018 | * | * | * | * | - | * | * | * | 7 |
| Kensuke Usuki | 2012 | * | * | * | * | * | * | * | * | 8 |
| Marie-Anne Hospital | 2014 | * | * | * | * | * | * | * | * | 8 |
| Adriano Venditti | 2019 | * | * | * | * | * | * | * | - | 7 |
| Eun-Ji Choi | 2021 | * | - | * | * | * | * | * | * | 7 |
| Xiao-Jun Huang | 2012 | * | * | * | * | - | * | * | * | 7 |
| Richard F. Schlenk | 2013 | * | * | * | * | * | * | * | * | 8 |
| Hong-Hu Zhu | 2013 | * | * | * | * | * | * | * | * | 8 |
| Wasithep Limvorapitak | 2018 | * | * | * | * | * | * | * | * | 8 |
| Meng | 2018 | * | * | * | * | - | * | * | * | 7 |

| | | | | | | | | | | |
|---|---|---|---|---|---|---|---|---|---|---|
| Lv | | | | | | | | | | |
| Adam Folta | 2019 | * | * | * | * | * | * | * | * | 8 |
| Nigel H. Russell | 2021 | * | * | * | * | * | * | * | * | 8 |
| Marilyn L. Slovak | 2000 | * | * | * | * | * | * | * | * | 8 |

**Note:**

The Newcastle-Ottawa Scale: Each study can get one point (*) if it meets one criterion, but it can get up to two points (* *) as for comparability.

**Supplementary Table 4a** The results of heterogeneity test for OS in the total population

| Treatment 1 | Treatment 2 | $i^2$.pair | $i^2$. network |
|---|---|---|---|
| Allo-SCT | Auto-SCT | 46.17 | 54.65 |
| Allo-SCT | Chemotherapy | 31.03 | 44.23 |
| Auto-SCT | Chemotherapy | 0 | 3.36 |
| Global I-squared | | 23.95 | 30.97 |

**Supplementary Table 4b** The results of heterogeneity test for OS in the low-risk population

| Treatment 1 | Treatment 2 | $i^2$.pair | $i^2$. network |
|---|---|---|---|
| Allo-SCT | Auto-SCT | 0 | 0 |
| Allo-SCT | Chemotherapy | 0 | 0 |
| Auto-SCT | Chemotherapy | 0 | 0 |
| Global I-squared | | 0 | 0 |

**Supplementary Table 4c** The results of heterogeneity test for OS in the intermediate-risk population

| Treatment 1 | Treatment 2 | $i^2$.pair | $i^2$. network |
|---|---|---|---|
| Allo-SCT | Auto-SCT | 56.98 | 58.51 |
| Allo-SCT | Chemotherapy | 34.14 | 40.45 |
| Auto-SCT | Chemotherapy | 0 | 0 |
| Auto-SCT | Haplo-SCT | - | 10.45 |
| Haplo-SCT | Chemotherapy | 73.22 | 65.52 |
| Global I-squared | | 46.89 | 46.70 |

**Supplementary Table 4d** The results of heterogeneity test for DFS in the total population

| Treatment 1 | Treatment 2 | $i^2$.pair | $i^2$. network |
|---|---|---|---|
| Allo-SCT | Auto-SCT | 61.42 | 78.97 |
| Allo-SCT | Chemotherapy | 50.97 | 90.48 |
| Auto-SCT | Chemotherapy | 0 | 68.54 |
| Global I-squared | | 38.64 | 53.91 |

**Supplementary Table 4e** The results of heterogeneity test for DFS in the low-risk population

| Treatment 1 | Treatment 2 | $i^2$.pair | $i^2$. network |
|---|---|---|---|
| Allo-SCT | Auto-SCT | 0 | 0 |
| Allo-SCT | Chemotherapy | 0 | 0 |
| Auto-SCT | Chemotherapy | 0 | 0 |
| Global I-squared | | 0 | 0 |

**Supplementary Table 4f** The results of heterogeneity test for DFS in the intermediate-risk population

| Treatment 1 | Treatment 2 | $i^2$.pair | $i^2$. network |
|---|---|---|---|
| Allo-SCT | Auto-SCT | 89.75 | 82.33 |
| Allo-SCT | Chemotherapy | 70.40 | 67.27 |
| Auto-SCT | Chemotherapy | 0 | 37.14 |
| Haplo-SCT | Chemotherapy | 61.88 | 61.95 |
| Global I-squared | | 72.99 | 74.00 |
